# Supplementary material for: Bio-Inspired Micromachined Volumetric Flow Sensor with a Big Dynamic Range for Intravenous Systems
Source: Sensors (Basel). 2022 Dec 26;23(1):234. doi: 10.3390/s23010234 (PMC9823585; doi:10.3390/s23010234)
Supplement: Supplementary file 1 [file sensors-23-00234-s001.zip › sensors-2083907-supplementary.pdf]

sensors

MDPI

Article

Bio-Inspired Micromachined Volumetric Flow Sensor with a Big Dynamic Range for Intravenous Systems

Lansheng Zhang [1], Yingchen Yang [2], Georgios A. Bertos [3,4,5], Chang Liu [6] and Huan Hu [1,7,*]

[1] ZJU-UIUC Institute, International Campus, Zhejiang University, Haining 314400, China; lansheng.20@intl.zju.edu.cn
[2] Department of Mechanical Engineering, University of Texas Rio Grande Valley, Edinburg, TX 78539, USA; yingchen.yang@utrgv.edu
[3] Department of Physical Medicine and Rehabilitation, Feinberg School of Medicine, Northwestern University Prosthetics Orthotics Center, Chicago, IL 60611, USA; george.bertos@gmail.com or gbertos@central.ntua.gr
[4] School of Mechanical Engineering, National Technical University of Athens, 15780 Athens, Greece
[5] Applied Sciences and Technology, Baxter Healthcare Inc., Round Lake, IL 60073, USA
[6] Institute of Electrics, Chinese Academy of Sciences, Beijing 100089, China; changliu8888@gmail.com
[7] State Key Laboratory of Fluid Power and Mechatronic Systems, Zhejiang University, Hangzhou 310027, China
* Correspondence: huanhu@intl.zju.edu.cn

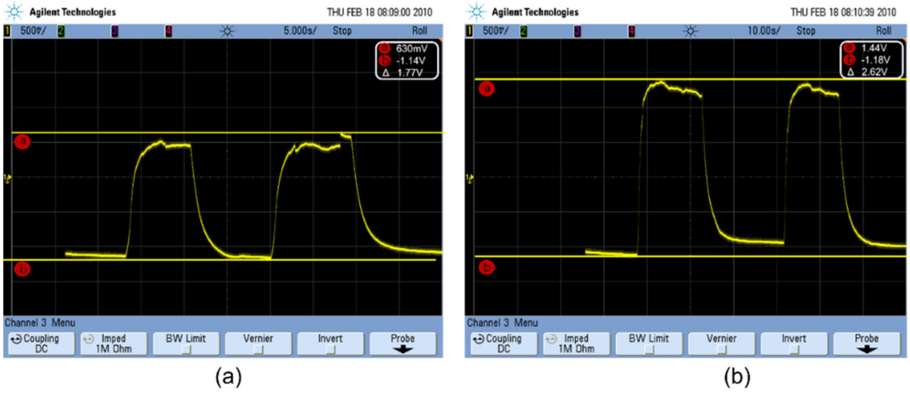

Figure S1. Oscilloscope screenshots show the sensor output at a volumetric flow rate of 100 mL/h. and (a) and 200 mL/h (b).

Table S1. The abbreviations used in this article and corresponding full names.

Abbreviation	Full name
MEMS	Micro-Electro-Mechanical System
IV	intravenous
AHC	artificial haircell
TSV	through-silicon via
PCB	printed circuit board
SOI	silicon-on-insulator
DRIE	deep reactive ion etching
BOE	buffered oxide etchant
IoT	Internet of Things
